# Clinical experience of an adhesive bone conduction hearing system in children with congenital single-sided deafness

**DOI:** 10.1016/j.bjorl.2024.101427

**Published:** 2024-03-25

**Authors:** Yujie Liu, Wenxi Qiu, Lin Yang, Yuan Wang, Jikai Zhu, Mengshuang Lv, Shouqin Zhao

**Affiliations:** a Capital Medical University, Beijing Tongren Hospital, Department of Otolaryngology Head and Neck Surgery, Beijing, China; b Capital Medical University, Ministry of Education Key Laboratory of Otolaryngology Head and Neck Surgery, Beijing, China

**Keywords:** Deafness, Paediatric single-sided deafness, Adhesive bone conduction device, Speech perception, Sound localisation

## Abstract

**Objectives:**

This study aimed to investigate the effects of an adhesive bone conduction device (aBCD) in children with congenital single-sided deafness (SSD). Specifically, we examined whether the aBCD elicits improvement in the speech perception ability of children with congenital SSD and whether using this device would adversely affect the horizontal localisation abilities of these children.

**Methods:**

Thirteen school-aged children with SSD and seven children with Normal Hearing (NH) were included in this study. Speech perception in noise was measured using the Mandarin Speech Test Materials and sound localisation performance was evaluated using broadband noise stimuli (0.5–20 kHz), randomly played from seven loudspeakers at different stimulus levels (65-, 70-, and 75-dB SPL).

**Results:**

All children with SSD showed inferior speech perception and sound localisation performance compared with children with NH. The aBCD use remarkably improved the speech perception abilities of these children under quiet and noise conditions; however, their sound localisation abilities neither improved nor deteriorated.

**Conclusion:**

This study reveals the effectiveness and safety of a non-surgical aBCD in paediatric patients with SSD. Our results provide a theoretical basis for early hearing intervention with an aBCD in children with congenital SSD who are temporarily unable to undergo ear surgery.

**Level of evidence:**

Level 3.

## Highlights

• Adhesive bone conduction device elicits improvement in speech perception.
• Adhesive bone conduction do not improve sound localisation abilities.
• Adhesive bone conduction is useful to children with single-sided deafness.

## Introduction

Single-sided deafness (SSD) is characterised by profound hearing loss in the affected ear and Normal Hearing (NH) in the contralateral ear [1], [2]. Because hearing basic speech signals through the healthy ear is possible in a quiet environment, the hearing problems of these patients are usually ignored. However, increasing evidence indicates that patients with SSD present with auditory deficits in speech perception under noise and sound localisation [3], [4]. Consequently, they may experience a lack of security, academic underachievement, and reduced quality of life.

Given the need for daily communication, hearing interventions for bilateral sensorineural hearing loss are standardised. However, there is no consensus on the treatment of patients with SSD. Current interventions for paediatric SSD include fitting Cochlear Implants (CIs), Bone Conduction Implants (BCIs), Contralateral-Routing-Of-Signal (CROS) hearing aids, and non-surgical Bone Conduction Devices (BCDs) [5], [6], [7], [8]. For patients with cochlear nerve deficiency, inner ear abnormalities, or immature anatomy, which may impede the implantation of CIs or BCIs, CROS hearing aids and non-surgical BCDs are useful alternatives. A CROS hearing aid collects sound signals from the affected ear and transmits them to an output transducer inserted in the canal of the ear with NH; hence, patients are required to wear two hearing aids simultaneously. A more aesthetically pleasing choice is a non-surgical BCD, which is attached to the mastoid region behind the impaired ear using a headband, soft-band, or an adhesive adapter, and it transmits sound signals to the ipsilateral cochlea [6], [9]. BCDs alleviate the Head Shadow Effect (HSE) to improve speech perception, especially for speech signals transmitted from the hearing field of the impaired ear. Notably, as BCDs do not provide actual auditory stimuli to the deaf ear, it is widely accepted that BCDs cannot improve the localisation abilities of patients with SSD [10], [11]. However, some adults with SSD can achieve horizontal directional hearing by learning to use ambiguous monaural cues (e.g. sound level and veridical spectral-shape cues) from the healthy ear [12], especially in familiar environments. Therefore, it is important to clarify whether BCDs will jeopardise patients’ original directional hearing ability [13].

In 2017, ADHEAR, a non-surgical solution based on adhesive BCDs (aBCDs), became available. The ADHEAR (MED-EL, Innsbruck, Austria) system consists of an adhesive adapter and an audio processor. The audio processor converts sound into mechanical vibrations and transmits the vibrations to the mastoid bone via the adhesive adapter, which is placed on the skin behind the auricle. The efficacy of ADHEAR for improving hearing in patients with conductive hearing loss has been widely reported, with accounts of more aesthetic satisfaction and less skin complications compared with conventional bone conduction hearing aids [14], [15]. However, clinical research on patients with SSD fitted with ADHEAR remains insufficient. To our knowledge, only two studies on the audiological outcomes of ADHEAR in adults with SSD (mean age, 40-years) have been published [6], [9]. Moreover, data on the performance of this new system in school-aged children and adolescents are lacking. This study, therefore, aimed to examine whether an aBCD elicits improvement in speech perception in school-aged children with congenital SSD. In addition, we compared the audiological outcomes of children with SSD with those of children with NH and assessed whether using an aBCD would negatively affect horizontal localization in children with SSD.

## Methods

### Participants

Thirteen school-aged children with SSD (age range, 5–12 years; mean, 7.46-years; Standard Deviation [SD = 2.22-years], Table 1) were recruited in our hospital. All participants had a Pure Tone Audiometry (PTA) threshold ≥ 90 dB HL across the standard audiometric frequencies from 500 Hz to 4000 Hz in the impaired ear and a normal PTA threshold ≤ 20 dB HL from 500 Hz to 4000 Hz in the healthy ear. Hearing loss was detected either incidentally or from neonatal hearing screening results. To identify the aetiology and exclude other possible inner ear diseases, all patients underwent imaging examinations, including computed tomography and magnetic resonance imaging before enrolment; eight patients had inner ear malformation. Seven children with NH, aged 6–12 years (mean, 9-years; SD = 2.16-years) were recruited as the comparison group. NH was defined as bilateral normal PTA thresholds ≤ 20 dB HL from 500 Hz to 4000 Hz.

**Table 1:** Demographic data of 13 patients with congenital SSD.

Participants	Sex	Age (years)	Side	Mean PTA_0.25‒4 kHz_ of the contralateral ear (dB HL)	
				PTA-BC	PTA-AC
P1	M	7	L	10	10
P2	F	6	R	7	7
P3	F	12	R	7	7
P4	M	11	R	9	9
P5	M	7	R	9	9
P6	M	5	R	9	9
P7	F	7	L	10	10
P8	M	10	L	8	8
P9	F	7	L	8	8
P10	M	5	L	9	9
P11	M	8	L	7	7
P12	M	6	L	9	9
P13	M	6	R	7	7

SSD, single-sided deafness; side, the side of SSD; PTA_0.25‒4 kHz_, Pure Tone Threshold Audiometry of 5 frequencies (0.25, 0.5, 1, 2, 4 kHz); AC, Air Conduction; BC, Bone Conduction; F, Female; M, Male; R, Right side; L, Left side.

In the 13 patients, we examined the sound field hearing threshold, speech perception in quiet, and binaural hearing effects. Five patients did not participate in the examination of sound localisation because of time constraints or lack of cooperation with the measurement process. Therefore, only seven patients (P1–7) underwent sound localization assessment.

### Experimental design

Speech perception (including speech perception in quiet and binaural hearing conditions) and sound localisation were assessed in children with congenital SSD (when unaided and aBCD-aided) and healthy controls.

### Settings

The experiment was conducted in a sound-attenuated audiometric booth with seven audiometric loudspeakers positioned 1 m from the centre of the participant’s head, spanning from +90° to −90° (at 30° intervals) on the horizontal plane. All patients were fitted with a non-surgical aBCD (ADHEAR), which was uniformly programmed with an omnidirectional microphone during all experimental procedures. The volume setting of the aBCD was determined based on the patient’s preference, and the optimal level remained constant throughout all experiments.

### Outcome measures

#### Sound field hearing thresholds and speech perception in quiet

Warble tones at frequencies of 0.5, 1, 2, and 4 kHz, presented from the front (0°, azimuth), were used to determine the hearing thresholds (in dB HL) in the sound field. Speech perception in quiet was assessed using the speech discrimination score (SDS, in %) of each participant. Disyllabic speech signals, presented by a male speaker, were selected from the Mandarin Speech Test Materials [16]. The healthy ears were covered in this test session with an earmuff and earplug applied to the external auditory canal.

#### Binaural hearing effects

The binaural summation effect, squelch effect, and HSE were measured using the speech reception threshold (SRT) of each participant, which was tested under a constant masker of Speech-Spectrum Noise (SSN) fixed at 65 dB SPL. The target disyllabic speech signals from the Mandarin Speech Test Materials began at 65 dB SPL and adaptively fluctuated in 2 dB intervals depending on the participant’s response. SRT was defined as the speech signal level at which the participant identified the disyllabic word correctly 50% of the time. We calculated the Speech-to-Noise rRatio (SNR) as the difference between speech stimuli levels and SSN.

We conducted tests in different spatial configurations as follows: 1) Binaural summation effect (S_0_N_0_): speech signals and SSN were presented from the front (0°, azimuth). 2) Binaural squelch effect (S_0_N_SSD_): speech signals were presented from the front (0°, azimuth), while SSN was presented from the SSD side (−/+90°, azimuth). 3) HSE (S_SSD_N_NH_): speech signals were presented from the SSD side (−/+90°, azimuth), while SSN was presented from the NH side (−/+90°, azimuth).

In patients with NH, SSN was uniformly presented from the left side for points (2) and (3).

#### Sound localisation

Participants were placed at the centre of the semicircle (radius: 1 m) formed by seven loudspeakers. Broadband noise (0.5–20 kHz), with a duration of 1 s, was randomly played at three different sound levels (65-, 70-, and 75-dB SPL). During the formal test, each loudspeaker randomly provided sound stimuli twice at each sound-level burst. After each presentation, participants were allowed to promptly indicate the orientation without any feedback information.

### Statistical analysis

$$MAE=\frac{\sum_{i=1}^{n}\left|\alpha \begin{array}{c} i\\ RESP \end{array}-\alpha \begin{array}{c} i\\ TARG \end{array}\right|}{n}$$

We assessed the accuracy of sound localisation under different conditions by calculating the Mean Absolute Error (MAE) using the above equation. α_RESP_ and α_TRAG_ denote the response and target azimuths (in degrees), respectively. For participants with an optimal localisation ability, MAE is 0. A paired *t*-test was used for the analysis of differences between the unaided (aBCD off) and aided (aBCD on) conditions for each participant, whereas an independent *t*-test was used for comparisons between groups; *p-*values < 0.05 were considered statistically significant. Statistical analyses and diagram drawings were performed using SPSS 26.0 and GraphPad Prism 8.0, respectively.

## Results

### Hearing thresholds and speech perception in quiet

**Fig. 1:**
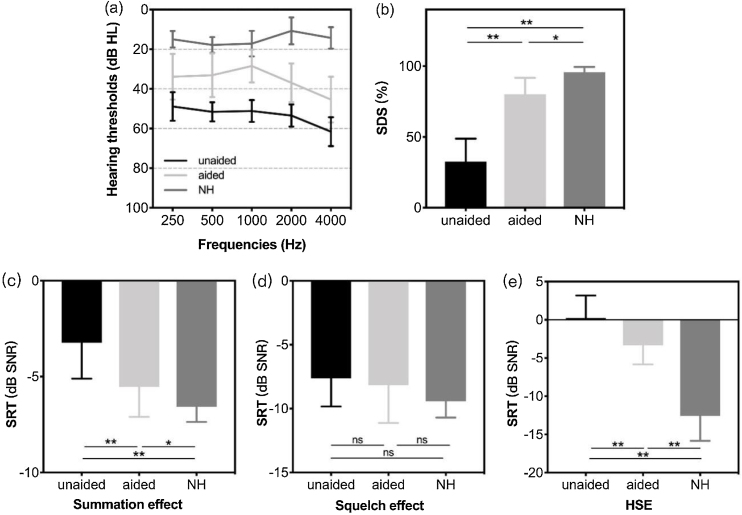
(a) Mean sound field hearing thresholds and (b) the mean speech discrimination score in a quiet environment in children with SSD (in unaided and aided conditions) and controls with NH. The speech reception threshold in noise for spatial configurations of (c) the summation effect (S_0_N_0_), (d) the squelch effect (S_0_N_SSD_), and (e) HSE (S_SSD_N_NH_). Group means are presented as mean ± two standard deviations. Significant differences are defined as *(*p* < 0.05), **(*p* < 0.01). SDS, speech discrimination score; NH, normal hearing; SSD, single-sided deafness; SRT, speech reception threshold; SNR, speech-to-noise ratio; HSE, head shadow effect; NH, normal hearing; ns, not significant.

The mean (SD) hearing thresholds of patients with SSD in the unaided (aBCD off) and aided (aBCD on) conditions were 53.31 (4.23) and 35.54 (9.26) dB HL, respectively, indicating an average functional gain of 17.77 (7.11) dB HL (*p* < 0.01); however, the mean aided hearing threshold was still inferior to that of children with NH (15 [4.58] dB HL, *p* < 0.01). The hearing threshold data of the SSD (aided and unaided) and NH groups for each frequency are presented in Fig. 1a. Compared to the unaided condition, the mean (SD) SDS in quiet improved from 32.54% (16.28%) to 80.31% (11.51%) with an aBCD (*p* < 0.01). However, the aided SDS in patients with SSD was still worse than that of the NH group (97.71% [2.43%]; *p* < 0.05); (Fig. 1b).

### Binaural hearing effects

Fig. 1(c–e) shows the SRTs of binaural effects in the different spatial configurations. For children with NH, the mean SRTs (SD) for the summation effect, squelch effect, and HSE were −6.57 (0.79), −9.43 (1.27), and −12.57 (3.26) dB SNR, respectively. For patients with SSD, the mean SRTs (SD) for the summation effect, squelch effect, and HSE in the unaided condition were −3.23 (1.88), −7.62 (2.22), and 0.23 (2.95) dB SNR, respectively. The corresponding mean SRTs (SD) in the aided condition were −5.54 (1.56), −8.15 (2.97), and −3.31 (2.53) dB SNR, respectively. There were significant differences between the SRTs for the summation effect and HSE in the unaided and aided conditions (all *p* < 0.01). In the spatial setting of the squelch effect, in which noise was presented from the impaired side, the aBCD did not improve speech perception (*p* = 0.407), which was within the expectations. There was an obvious discrepancy in the summation effect (*p* < 0.05) and HSE (*p* < 0.01) between the children with NH and those who used the aBCD.

### Sound localisation abilities

**Fig. 2:**
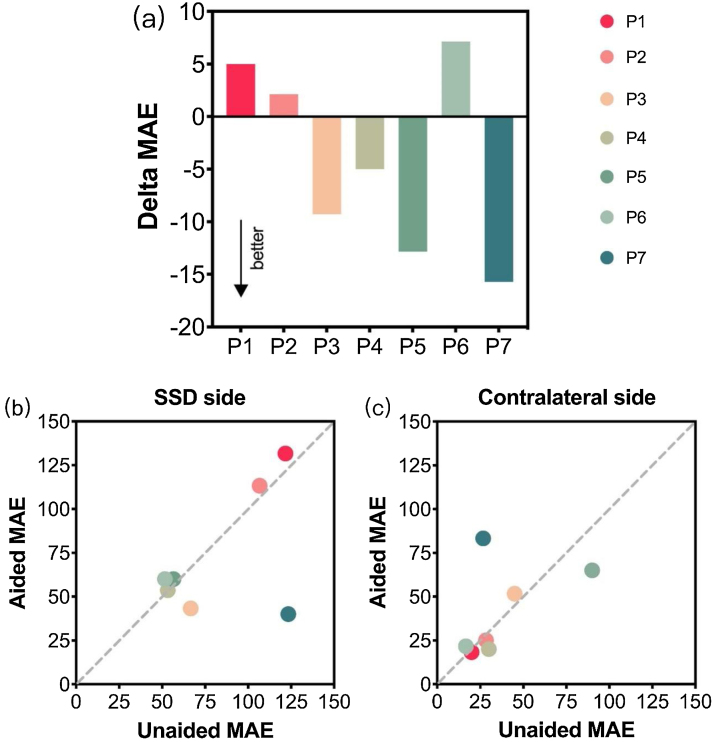
(a) Delta MAE values for each patient in the unaided and aided conditions. MAEs of unaided conditions (x-axis) are plotted against those of aided conditions (y-axis) and mean absolute errors are calculated on the (b) SSD side and on the (c) contralateral NH side. MAE, mean absolute error; P, patient; SSD, single-sided deafness; NH, normal hearing.

A comparison of the individual changes in sound localization (Fig. 2a) shows negative delta MAE for P3, P4, P5, and P7, indicating that their sound localization performance improved with the aid of the aBCD. However, the mean MEA for all patients in the unaided conditions was 58.77° (17.11°), which changed to 54.7° (15.1°) when patients used an aBCD; statistical analysis revealed no significant difference between the two conditions (*p* = 0.276). All individual scores are shown in Table 2.

**Table 2:** MAEs for patients (P1‒7) with congenital SSD and listeners with NH (N1‒7).

Participants	Unaided MAE (°)	Aided MAE (°)
P1	68.57	73.57
P2	67.86	70
P3	55.71	46.43
P4	39.29	34.29
P5	70.71	57.86
P6	32.14	39.29
P7	77.14	61.43
N1	0	‒
N2	0	‒
N3	0	‒
N4	0	‒
N5	0	‒
N6	0	‒
N7	0	‒

MAE, mean absolute error; SSD, single-sided deafness; NH, normal hearing.

To further determine whether the use of an aBCD would jeopardise the original localisation abilities of a patient, especially in the ear with NH, the MAEs for both sides were calculated. Fig. 2b–c shows the plots of the MAE values for the unaided condition versus those for the aided condition. Data points below the diagonal demonstrate an improvement in sound localization in the aided condition compared with the unaided condition. Overall, no significant differences were found on either side between conditions (SSD side: unaided MAE vs. aided MAE, 82.86° [32.92°] vs. 71.72° [35.91°], *p* = 0.416; contralateral side: unaided MAE vs. aided MAE, 36.67° [25.18°] vs. 40.71° [26.02°], *p* = 0.689), which suggests that the use of aBCDs did not have an obvious effect on sound localisation accuracy in the contralateral ear.

## Discussion

The high prevalence of inner ear malformation in children with SSD [17] necessitates the consideration of fitting of non-surgical aBCD as a vital hearing reconstruction strategy. Our results demonstrated that aBCDs considerably improved speech perception in children with SSD without worsening their original localisation abilities. To our knowledge, this is the first study assessing the effects of aBCDs in paediatric patients with SSD.

To avoid clinical heterogeneity among tests, the design of the audiological tests performed in the present study was based on the SSD testing framework published in 2016 [18]. In this study, children who used an aBCD showed lower response thresholds in the summation effect and HSE tests (all *p* < 0.01), indicating improved speech perception in a challenging environment. This promising result is consistent with those of some previous studies [8], [10], [19], [20], [21], [22] in which speech tests with competing noise were also performed on patients with SSD fitted with various BCDs. However, in the squelch effect test, the noise masker at the affected side provided less favourable SNRs, which may indicate some degradation of the speech perception of patients with SSD using an aBCD. Significant degradation of the response thresholds in patients with SSD fitted with BP100 hearing aids has been previously reported [23]. Similar to the majority of related studies [20], [24], our study found that the use of the aBCD did not substantially degrade the overall speech perception in noise.

There was an obvious discrepancy in the thresholds for the summation effect and HSE between the aided SSD and control groups. For patients with SSD, sufficient bone conduction signals are necessary to stimulate the contralateral cochlea, which is associated with a certain pressure caused by fitting the device [25]. The HSE contributes to the underdeveloped ability of patients with SSD to recognise speech in noise. Therefore, low transcranial attenuation is beneficial as it allows the conduction of more sound signals to the functional contralateral cochlea [26]. The aBCD attached to the mastoid of the impaired ear acts as a bone conduction signal transducer that transmits these signals from the affected side to the contralateral ear with NH. Consequently, it reduces the HSE to improve the SNR in the healthy ear and broadens the range of hearing perception. However, this does not provide sufficient binaural cues for patients with SSD to show speech recognition abilities similar to those of their peers with NH.

It has been demonstrated that some patients with unilateral hearing loss have relatively good directional hearing, especially in familiar environments [27], [28]. In this study, most patients with SSD showed poor horizontal-localisation performance (gain < 0.75), whereas the age-matched NH group showed perfect localisation performance in the same test procedure. This is inconsistent with a previous report of good sound localisation performance shown by some patients with SSD [29]. In that study, adults with SSD (age range: 17–68 years) learned to use monaural cues (e.g., spectral pinna cues) and HSE to maintain relatively good directional hearing. As monaural localisation takes a long time to develop, the monaural localisation development of the children in this study (average age: 9 [3.65] years) may have been immature; therefore, they showed worse localisation accuracy than the adults with SSD in the previous study.

Because BCDs do not provide actual binaural hearing for patients with SSD, using them may not improve their sound localisation abilities. This opinion was confirmed by our results and those of previous studies [5], [8], [9], [20], [30], which indicated no substantial differences in localisation performance between adults with SSD with and without BCDs. Grantham et al. reported significant deficits in localisation performance after the use of a BCD; however, this result was insignificant owing to the small sample size [13]. Although most studies revealed no obvious benefits for adults with SSD in using BCDs in audiological tests, higher subjective satisfaction with spatial hearing is encouraging. Huber et al. used the “Speech, Spatial, and Qualities of Hearing” questionnaire to evaluate the subjective benefits of BCDs in 17 adults with SSD and reported an improvement in the spatial hearing subscale in the aided condition: patients reported having better lateralisation (the ability to locate a speaker to the right or the left) after their devices were implanted [11]. Considering these promising findings, we believe that aBCDs do not worsen the intrinsic localisation ability of paediatric patients with SSD.

The efficacy of transdermal Bone Conduction Hearing Implants (BCHI) for SSD has been proven in previous studies [31], and the results of this study further confirm the effectiveness of BCDs in treating SSD.

In our study, all patients with single-sided deafness (SSD) had normal hearing in the contralateral ear, with hearing thresholds ≤ 20 dBHL. Will patients still benefit from BCDs if they also have impaired hearing in the contralateral ear, resulting in Asymmetric Hearing Loss (AHL)? Previous research has shown that bone conductive implantation can decrease the hearing thresholds and improve the quality of life for AHL patients [32], [33]. This suggests that AHL patients can also benefit from BCDs.

Previous studies have shown that cochlear implantation is also an effective method for treating SSD [34]. However, for patients with ossified cochlear, the anatomical structures of these patients’ cochlears have changed, which makes the cochlear implantation become an infeasible option, and BCDs may become the sole method for improving their hearing.

One important limitation was that subjective satisfaction could not be assessed because we only tested the short-term influence of ADHEAR systems. In addition, the sample size was small owing to the low availability of patients, and some patients were unwilling to use a BCD. Further studies with a larger sample of patients and subjective evaluations are imperative for yielding a more comprehensive presentation of spatial hearing outcomes. Moreover, longitudinal studies are needed to examine whether the limited benefits of BCDs for sound localisation performance can be improved in the long-term.

## Conclusion

Overall, our findings confirmed that children with SSD have a worse speech perception ability and worse sound localisation than children with NH. The fitting of non-surgical aBCDs was a useful hearing reconstruction strategy for improving speech perception in challenging environments without affecting the original sound localisation ability of the patient. These results provide a theoretical basis for early hearing intervention in children with SSD and the prognosis of subsequent bone conduction implantation.

## Data availability statement

All data generated or analysed during this study are included in this article and its supplementary material files. Further enquiries can be directed to the corresponding author.

## Funding

The study was supported by the 10.13039/501100001809National Natural Science Foundation of China (Grant nº 81770989) and the Capital Health Research and Development of Special (Grant nº 2020-2-2057) through Professor Shouqin Zhao.

## Conflicts of interest

The authors declare no conflicts of interest.

## CRediT authorship contribution statement

**Yujie Liu:** Conceptualization, Data curation, Methodology, Writing – original draft. **Wenxi Qiu:** Data curation, Formal analysis, Writing – review & editing. **Lin Yang:** Methodology, Investigation. **Yuan Wang:** Visualization. **Jikai Zhu:** Visualization, Writing – review & editing. **Mengshuang Lv:** Writing – review & editing. **Shouqin Zhao:** Conceptualization, Funding acquisition, Project administration.
